# Cathepsin L activated by mutant *p53* and Egr-1 promotes ionizing radiation-induced EMT in human NSCLC

**DOI:** 10.1186/s13046-019-1054-x

**Published:** 2019-02-07

**Authors:** Wenjuan Wang, Yajie Xiong, Xinyuan Ding, Long Wang, Yifan Zhao, Yao Fei, Ying Zhu, Xiao Shen, Caihong Tan, Zhongqin Liang

**Affiliations:** 1 Department of Pharmacology, College of Pharmaceutical Sciences, Soochow University, Suzhou, 215000 China; 2 Department of Pharmacy, Children’s Hospital of Soochow University, Suzhou, 215000 China; 3 Institutes for Translational Medicine, Soochow University, Suzhou, 215000 China; 4 Department of Pharmacy, The Affiliated Suzhou Hospital of Nanjing Medical University, Suzhou, 215000 China; 5 Department of Pharmacy, Affiliated Hospital of Jiangsu University, Zhenjiang, 212000 China

**Keywords:** Non-small cell lung cancer, Ionizing radiation, Epithelial-mesenchymal transition, *p53* mutation, Cathepsin L

## Abstract

**Background:**

Ionizing radiation (IR) is one of the major clinical therapies of cancer, although it increases the epithelial-mesenchymal transition (EMT) of non-small cell lung cancer (NSCLC), unexpectedly. The cellular and molecular mechanisms underlying this role are not completely understood.

**Methods:**

We used NSCLC cell lines as well as tumor specimens from 78 patients with NSCLC to evaluate *p53,* Cathepsin L (CTSL) and EMT phenotypic changes. Xenograft models was also utilized to examine the roles of mutant *p53* (*mut-p53*) and CTSL in regulating IR-induced EMT of NSCLC.

**Results:**

Expression of CTSL was markedly increased in human NSCLC tissues with mutant *p53* (*mut-p53*), and *p53* mutation positively correlated with metastasis of NSCLC patients. In human non-small cell lung cancer cell line, H1299 cells transfected with various *p53* lentivirus vectors, *mut-p53* could promote the invasion and motility of cells under IR, mainly through the EMT. This EMT process was induced by elevating intranuclear CTSL which was regulated by *mut-p53* depending on Early growth response protein-1 (Egr-1) activation. In the subcutaneous tumor xenograft model, IR promoted the EMT of the cancer cells in the presence of *mut-p53*, owing to increase expression and nuclear transition of its downstream protein CTSL.

**Conclusion:**

Taken together, these data reveal the role of the *mut-p53*/Egr-1/CTSL axis in regulating the signaling pathway responsible for IR-induced EMT.

**Electronic supplementary material:**

The online version of this article (10.1186/s13046-019-1054-x) contains supplementary material, which is available to authorized users.

## Background

Lung cancer is the most lethal cancer worldwide, and approximately 80% of lung cancers are non-small cell lung cancer (NSCLC) [1]. Radiation therapy is one of the major clinical tools of NSCLC treatment, together with chemotherapy and surgery [2]. Radiotherapy causes DNA damage directly by ionization, thereby destroying cancer cells. However, recent studies indicated that ionizing radiation (IR), paradoxically, promotes invasion and metastasis of NSCLC cells by inducing the epithelial-mesenchymal transition (EMT) [3, 4]. Invasion and metastasis are the main obstacles to successful therapy and are closely linked to the mortality rate of NSCLC. Therefore, the mechanism of IR-induced EMT in NSCLC is needed to be elucidated urgently.

The progress of NSCLC involves multiple genetic abnormalities that lead to EMT of the aggressive bronchial epithelial cells [5, 6]. Among such genetic abnormalities, *mut-p53* occurs in about 50% of NSCLC [7]. Apart from the loss of tumor-suppressor functions, *mut-p53* may gain new functions independent of wild-type *p53* (*wt-p53*), defined as gain-of-function (GOF), which enable them to contribute to malignant progression, including increased cell invasion and metastasis which are the main traits of EMT [8, 9]. Interestingly, patients whose tumors contain *p53* gene present an increase in tumor metastasis when underwent radiation or DNA-damaging reagents [10]. However, a few reports have shown *p53* mutation as a delayed effect of radiation, and the correlation between *mut-p53* and IR-induced EMT in NSCLC is scarcely known.

Our previous study showed that IR promoted EMT especially in *mut-p53* human glioma cells, and the key effector that induces EMT may be Cathepsin L (CTSL) [11]. CTSL, a ubiquitously expressed lysosomal cysteine protease, is primarily involved in terminal degradation of intracellular and endocytosed proteins [12]. Accumulating evidences reveal that CTSL specifically high-expressed in a wide range of human cancers [13–16]. Simultaneously, our recent study indicated that the expression level of CTSL correlates positively with the degree of tumor malignancy [14]. Moreover, CTSL transported into the nucleus plays an important role in regulating cellular transcription factors, and thus affects the morphology or activity of cancer cells. Notably, the nuclear CTSL activates the transcription of EMT genes and also confers a replicative and metastatic advantage to tumor cells [13]. In fact, we also found that CTSL inhibition could suppress EMT-mediated invasion and metastasis of lung cancer cells [17]. Overall, the role of CTSL in promoting tumor progression and metastatic aggressiveness have raised significant interest in the upstream genes of CTSL intervention strategies. Indeed, one research reported that human CTSL promoter contains two *p53* binding motifs and *wt-p53* could upregulate CTSL in a static state [18]. However, there was no report which explains the role of *mut-p53* in regulating CTSL and the pathway of *mut-p53* functions. The fact that *mut-p53* facilitates transcription of transformation related genes was shown in several studies [19]. Of note, induction of Early growth response-1 (Egr-1) gene expression was one of the significant changes observed upon overexpression of *mut-p53* in human lung cancer cells [20]. The Egr-1 protein is a transcription factor involved in various biological functions including regulation of proliferation, differentiation and apoptosis [21]. It is noteworthy that Egr-1 is a direct transcriptional target of *mut-p53* which demonstrated by promoter analysis and chromatin immunoprecipitation assays. Both Egr-1 mRNA and Egr-1 protein levels were upregulated by *mut-p53.* Interestingly, Egr-1 is not only participates in the GOF effect of *mut-p53* but also an IR responsive factor [22]. Furthermore, this stress response gene is involved in CTSL expression regulation under both genetic and metabolic stress conditions in human carcinoma cells [23]. These data suggest that specific regulation of Egr-1 binding on CTSL promoter could be triggered under conditions others than normal culture conditions. In addition, it was described that Egr-1 also showed a different function during EMT in NSCLC [24]. Collectively, these findings show that Egr-1 may be the target protein of *mut-p53* in regulating CTSL mediated EMT under IR.

Herein, we consequently proposed a hypothesis that the transcriptional activation of CTSL which was mediated by *mut-p53/*Egr-1 axis increases the IR-induced EMT. Besides, we have also indicated that expression of CTSL correlates with radioresistant, as well as malignant grades of tumor tissues [14]. Thus, on the basis of our previous findings, it is of scientific significance to study the mechanism of *mut-p53* up-regulating CTSL expression and promoting IR-induced EMT of NSCLC cells. Overall, our study defines CTSL as a novel target of *mut-p53* under IR and provides new insights for the understanding of *mut-p53* GOF activity.

## Methods

### Patients and tumor specimens

A cohort of 78 diagnosed patients with NSCLC was treated at the Affiliated Hospital of Jiangsu University (Zhenjiang, China) between March 2012 and February 2013 to be included in this study. Informed consent from all patients was obtained according to the Declaration of Helsinki, and the studies were approved by decisions of the Ethics Committee of Jiangsu University. Tumor specimens and adjacent tissue samples were immediately snap-frozen in liquid nitrogen after surgical removal. All the specimens were collected for genetic mutation analysis, western blotting and immunohistochemistry assays. The histopathological assessment was carried out separately by two pathologists and ended with a consensus decision. The staging system was carried out according to the 2009 IASLC classification. Brief clinical and pathologic characteristics of the subjects are presented in Additional file 1**:** Table S1. Besides clinicopathological characteristics, we also collected follow-up information in a subgroup of 60 patients, including treatment, survival status and survival time. Among them, there were 12 patients (20.0%) receiving radiotherapy. The survival time of these patients ranged from 3 to 60 months with the median of 42.9 months.

### Cell line and culture conditions

Human NSCLC cell lines (H1299, H1650, VMRC-LCD, H322, and H1838) were purchased from the Type Culture Collection of the Chinese Academy of Sciences (Shanghai, China) and cultured in RPMI-1640 (Gibco) supplemented with 10% fetal bovine serum (FBS, Gibco Life Technologies), 100 unit/ml penicillin and 100 units/ml streptomycin. The cells were grown in a humidified incubator with 5% CO_2_ at 37 °C.

### IR condition

The human and mice cells were irradiated using a 6-MV X-ray linear accelerator (model: PRIMUS, D. E., Siemens, Malvern, PA, USA) at a dose rate of 198 cGy/min. After IR, the cells were placed in the incubator and samples were collected at the indicated time points (0, 6, 12, 24 h).

### Genetic mutation analysis

Total RNA was extracted from the fresh-frozen fraction of tumor tissue using Trizol reagent (Invitrogen). RNA samples were then used to synthesize cDNA using a PrimeScript RT reagent Kit (Takara) following the manufacturer’s instructions. Primer sequences (Additional file 2**:** Table S2) were designed for mutational analysis of exons 5 to 8 of the *p53* gene. PCR product sizes were controlled by migration on 2% agarose electrophoresis gel and were purified with DNA gel extraction kit (Axygen). Targeted exon sequences were verified by Sangon Biotech (Shanghai, China).

### Immunohistochemistry

The ABC detection system (Vectastain Elite ABC Kit, Vector) was used for immunostaining according to the manufacturer’s protocol as described previously [13]. Specific primary antibodies were used as follows: anti-CTSL (Abcam, ab6314), anti-Snail (Santa Cruz, sc-28199), anti-E-cadherin (Abcam, ab1416), anti-N-cadherin (Abcam, ab18203) and anti-Vimentin (Abcam, ab92547). The negative control slides, without the primary antibodies, did not exhibit nonspecific staining. In this study, all slides were examined by two pathologists in a blinded fashion. To evaluate the expression level of the proteins, the intensity of staining was scored according to a positive index of tumor cells.

### Western blotting

Western blotting was conducted as previously described [25]. The primary antibodies used in this study are as follows: anti-CTSL (Abcam, ab6314), anti-p53 (Abcam, ab1101), anti-E-cadherin (Abcam, ab1416), anti-N-cadherin (Abcam, ab18203), anti-Vimentin (Abcam, ab92547), anti-Zeb1 (Abcam, ab203829), anti-Snail (Santa Cruz, sc-28199), anti-Egr-1 (Santa Cruz, sc-189), and loading control anti-β-actin (Abcam, ab8226). Finally, the primary antibodies were probed with rabbit or mouse secondary antibodies labeled with DyLight 800 (KPL) and scanned with the Odyssey Infrared Imaging System (LI-COR).

### Construction of point mutation plasmids

The p3XFLAG-Myc-CMV-*p53* (Flag-p53) expression vector, which carries wild-type *p53*, was a generous gift from Professor Wang Guanghui at the Laboratory of Molecular Neuropathology, College of Pharmaceutical Sciences, Soochow University. Flag-p53 (R175H), Flag-p53 (R248Q), and Flag-p53 (R273H) expression vectors, containing *p53-R175H*, *p53-R248Q*, and *p53-R273H* mutants, were generated from Flag-p53 by a MutanBEST Kit (TaKaRa) following the manufacturer’s recommendations. The primers sequences used for mutagenesis were listed in Additional file 3**:** Table S3. The sequences of the mutation constructs were verified by Sangon Biotech (Shanghai, China). Transfection of these plasmids was performed using Lipofectamine® 2000 Reagent (Invitrogen) according to the manufacturer’s instructions as previously described [26].

### Lentivirus transfection and isolation of stable cell clones

The *p53*-null H1299 cells were transfected separately with 2 × 10^7^ titration units of lentivirus packaging (Flag lentivirus, Flag-p53 lentivirus, and Flag-p53 (*R273H*) lentivirus) synthesized by Gene Chem (Shanghai, China). After 2 days of transfection, the medium was replaced with fresh RPMI-1640 containing 10% FBS and 300 μg/mL G418 (Roche). After the dilution culture was limited under the pressure of G418, several independent clones of each transfection group were selected and screened for p53 protein expression by western blotting. Three stable transfected cell clones were selected for further experiments and each designated as Flag, Flag-p53, and Flag-p53 (R273H).

### Immunofluorescence staining

Cells were grown on glass coverslips (Fisher Scientific). After treatment with 8 Gy of IR, cells were fixed with 4% paraformaldehyde. Following the PBS (phosphate buffer saline) wash, cells were permeabilized using 0.2% Triton X-100, incubated in a blocking solution (PBS, 3% BSA (bovine serum albumin)), and further incubated overnight at 4 °C with primary antibodies, including anti-CTSL (Abcam, ab6314), anti-E-cadherin (Abcam, ab1416), anti-N-cadherin (Abcam, ab18203) and anti-Vimentin (Abcam, ab92547). Thereafter, Alexa Fluor 488 (Molecular Probes, 1:500) was added as a fluorescent-conjugated secondary antibody. For F-actin staining of the cytoskeleton, cells were stained with FITC-Phalloidin (Thermo Fisher Scientific). DAPI (Sigma Aldrich) was used as a nuclear counterstain. Finally, the coverslips were mounted onto slides with fluorescent mounting medium (Vector Labs) and immediately observed by confocal microscopy (Carl Zeiss, LSM 710).

### Wound-healing assay

For the wound-healing assay, the cells were grown in 6-well plates. When a confluent layer was achieved, the cells were scratched using a pipette tip, rinsed to remove debris, and then further incubated with fresh culture medium containing 1% fetal bovine serum (FBS). The initial distance of the wound was recorded at the beginning in order to estimate the recovery of the wounded layer by cell migration, after 24 h the images were captured using an inverted phase-contrast microscope. The wound-healing index, which was determined as a percentage, was quantitatively analyzed using 20 randomly selected distances across the wound from 0 h to 24 h, divided by the distance measured at 0 h. For each experiment, three replicates were performed.

### Cell invasion assay

Cell invasion assay was performed using a Boyden chamber containing 24-well transwell plates (Corning) with 8 μm pores on the membrane. All experiments were performed in duplicate and repeated three times. The membrane was coated with 100 μl 1:8 diluted Matrigel (BD Biosciences). After solidifying Matrigel at 37 °C, approximately 1 × 10^5^ cells in 200 μl culture medium supplemented with 1% FBS were seeded into the upper chamber, whereas the lower chamber was filled with the complete medium. Subsequently, the Boyden chamber was incubated at 37 °C with a 5% CO_2_ atmosphere for 24 h. On the assessment day, cells from the interior of the inserts were removed and the membranes were stained with crystal violet. The cells were counted by photographing the membrane through the microscope.

### Chromatin immunoprecipitation (ChIP) assay

Chromatin immunoprecipitation was carried out by using the EZ-ChIP Chromatin Immunoprecipitation Kit (Millipore) according to the manufacturer’s protocol. Briefly, cells were fixed with 1% formaldehyde at room temperature for 10 min to cross-link proteins to DNA. Then, the cross-linked chromatin was sonicated into 200 to 1000 bp fragments and immunoprecipitated using anti-p53 (Abcam, ab1101), anti-Egr-1 (Santa Cruz, sc-189) or IgG control antibodies. The samples were extensively washed after the cross-links were reversed and DNA was purified. The primers used for PCR amplification of specific promoter regions were listed in Additional file 4**:** Table S4.

### RNA interference

CTSL siRNA (Santa Cruz, sc-29938) and Egr-1 siRNA (Additional file 5**:** Table S5) were obtained from GenePharma (Shanghai, China). For transfection, siRNA was mixed with Lipofectamine® 3000 Reagent (Invitrogen) and then transfected into Flag-p53 (R273H) cells. The entire process was conducted according to the manufacturer’s guidelines. After 6 h, the supernatant was replaced with fresh medium containing 10% FBS and cultured for another 24 h.

### Animal experiments

All mice experiments were conducted in accordance with the humane treatment of animals under institutional guidelines approved by the Ethical Committee of Soochow University. The mice were housed in individually ventilated cages in the Animal Laboratory of the Medical College of Soochow University. Five-week-old male BALB/c nude mice (Animal experiment Center of Soochow University, Suzhou) were used in the study. Subcutaneous tumor transplantation was conducted using the Flag, Flag-p53, and Flag-p53 (R273H) cells. Cells (*n* = 1 × 10^7^) were resuspended in 100 μl PBS and implanted into the right flank of nude mice under sterile conditions. After the formation of palpable tumors (tumor volume reached 100 mm^3^), mice were randomized into six groups (5 mice per group): Flag group, Flag plus IR group, Flag-p53 group, Flag-p53 plus IR group, Flag-p53 (R273H) group, and Flag-p53 (R273H) plus IR group. The size of the tumor and the body weight of each mouse were measured as described previously [14]. The IR treatment was given quartic with a dose of 5 Gy until 20 Gy IR had been delivered. Mice were sacrificed on day 20, and tumor tissues were harvested for western blotting and immunohistochemistry.

### Statistical analysis

All quantitative data are presented as mean ± S.D. from at least three independent experiments per data point. Statistical analyses were performed using GraphPad Prism version 5.0 (GraphPad Software). Quantitative variables were analyzed with the Student’s *t*-test. The chi-square (*χ*^2^) test or Fisher exact test was used to compare qualitative variables. The overall survival (OS) of NSCLC patients was estimated using the Kaplan-Meier method. Mantel-Cox’s proportional hazard regression model was established for univariate and multivariate analyses of the combined contribution of *p53* and clinicopathological features to the OS of patients. *P* < 0.05 was considered statistically significant.

## Results

### *p53* mutation promoted metastasis and reduced the survival time of NSCLC patients

**Fig. 1:**
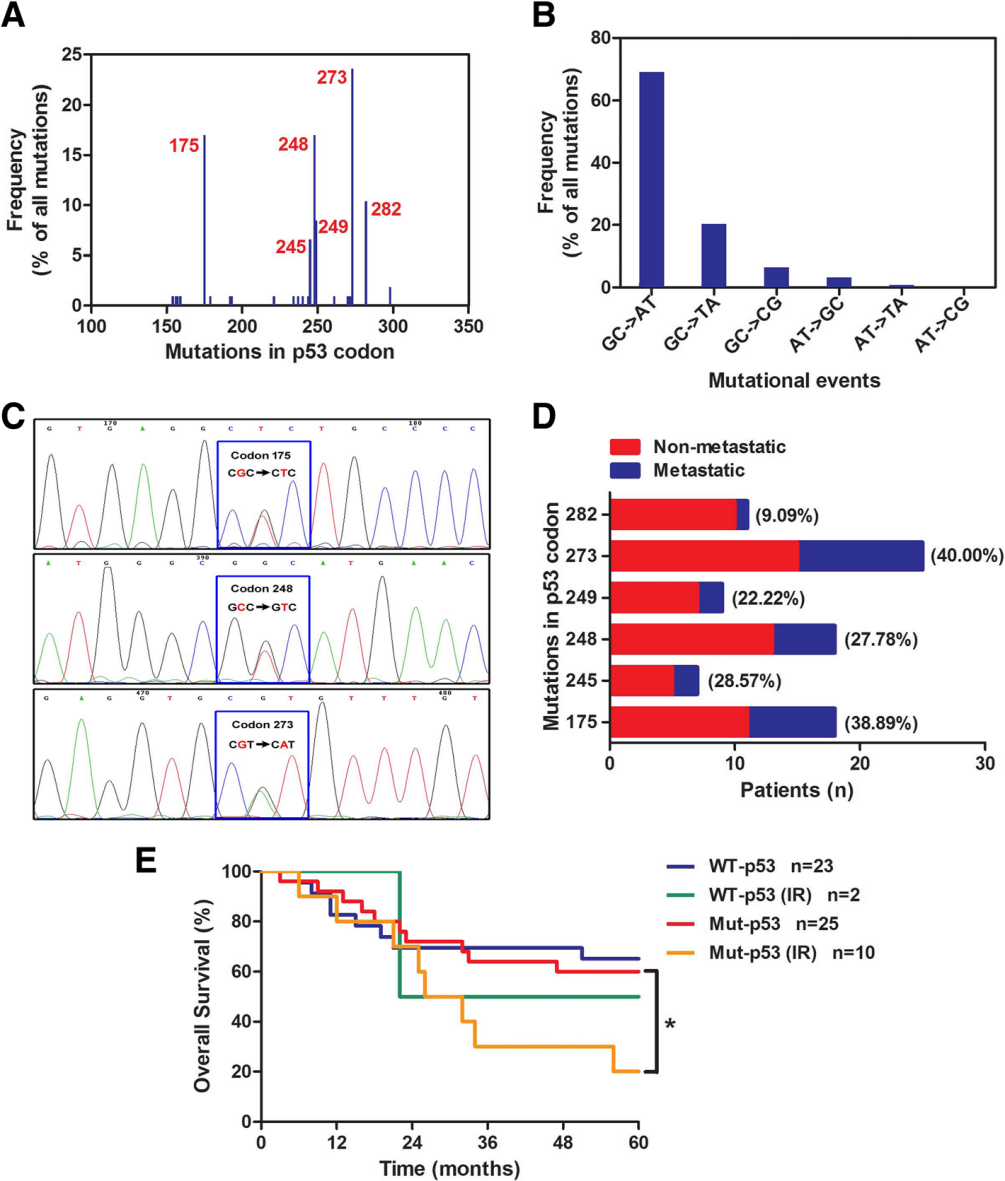
*p53* mutation promoted metastasis and reduced the survival time of NSCLC patients. **a**) The mutation frequency of *p53* codons in 78 NSCLC tissues. The mutational events of *p53* codons **b**) and representative mutation **c**) were shown. **d** Analysis of *p53* mutation in metastatic (lymph node metastasis and distant organ metastasis) and non-metastatic tissues from human lung cancer. **e** Correlation of *p53* (*wt-p53* and *mut-p53*) with or without radiotherapy and the overall survival time of the NSCLC patients were shown. Survival curves were constructed using the Kaplan-Meier method and analyzed by the log-rank test. *, *P* < 0.05

As a first step to investigate the relationship between *p53* mutant status and the clinicopathologic features of lung cancer patients, the statistic analysis of clinicopathologic characteristics in 78 NSCLC patients was showed the *mut-p53* was both positively related to the nodes metastasis (*P* = 0.0267) and TNM stage (*P* = 0.0399) (Table 1). Further, PCR amplification following direct sequencing was used to detect *p53* gene in 78 NSCLC tumor tissues. The result showed that the *p53* gene existed in all tumor tissues. Overall, *p53* point mutations detected in this study occurred at 6 major codons (Fig. 1a): codons 175 (16.98%), 245 (6.60%), 248 (16.98%), 249 (8.49%), 273 (23.58%), and 282 (10.38%). The mutations accounted for 55.13% (43 of 78) of all point mutations observed, and these hot spots corresponded to other reports on NSCLC tumors [27–30]. The main patterns of *p53* mutation were GC to AT transitions (69.10%) and GC to TA transversions (20.30%), which were strongly indicating the exposure to exogenous carcinogens (Fig. 1b and Fig. 1c). Different mutant codons of *p53* gene present various biological functions, and it is scarcely possible to accurately regulate *mut-p53* for achieving the effective therapy of multiple diseases. Figure 1d analyzed the tumor metastasis (lymph node metastasis and distant organ metastasis) rate of point mutations of *p53*. It was shown that the tumor metastasis rates of mutated *p53* at codon 175 (38.89%), codon 248 (27.78%) and codon 273 (40.00%) were more frequent. In addition, Kaplan-Meier analysis revealed that irradiated *mut-p53* NSCLC patients (*n* = 10) showed a poor prognosis compared with an irradiated *wt-p53* group (*n* = 2), although there was no statistical significance, mainly due to the restricted amount of irradiated *wt-p53* samples (Fig. 1e). Most notably, IR markedly reduced the survival time of *mut-p53* NSCLC patients (*P* = 0.0490). Thus, our data indicated that mutations of *p53* at codon 175, codon 248 and codon 273, may be the key of metastasis in NSCLC patients, and radiotherapy in patients with *mut-p53* predict more malignant characteristics.

**Table 1:** The correlation between *p53* mutational status and clinicopathologic features in 78 NSCLC patients

Clinicopathological feature	*p53*		χ ^2^	*P*-value
	WT (*n* = 35)	Mut (*n* = 43)		
Gender			1.4110	0.2349
Male	19	29		
Female	16	14		
Age (years)			0.2276	0.6333
< 60	12	17		
≥60	23	26		
Histological type			4.2338	0.1165
SCC	8	17		
ADC	27	26		
Differentiation			3.8980	0.1424
well	5	4		
Moderate	24	23		
poor	6	16		
Nodes metastasis			4.9079	0.0267^*^
N0	25	20		
N1-3	10	23		
Distant metastasis			–	0.1232
M0	35	39		
M1	0	4		
TNM stage			6.4440	0.0399^*^
I	16	15		
II	7	12		
III-IV	12	16		

*P* by χ ^2^ test, squamous cell carcinoma (SCC), adenocarcinoma (ADC)

*P < 0.05 was considered significant

### IR promoted cell motility and EMT in the presence of *mut-p53* in vitro

**Fig. 2:**
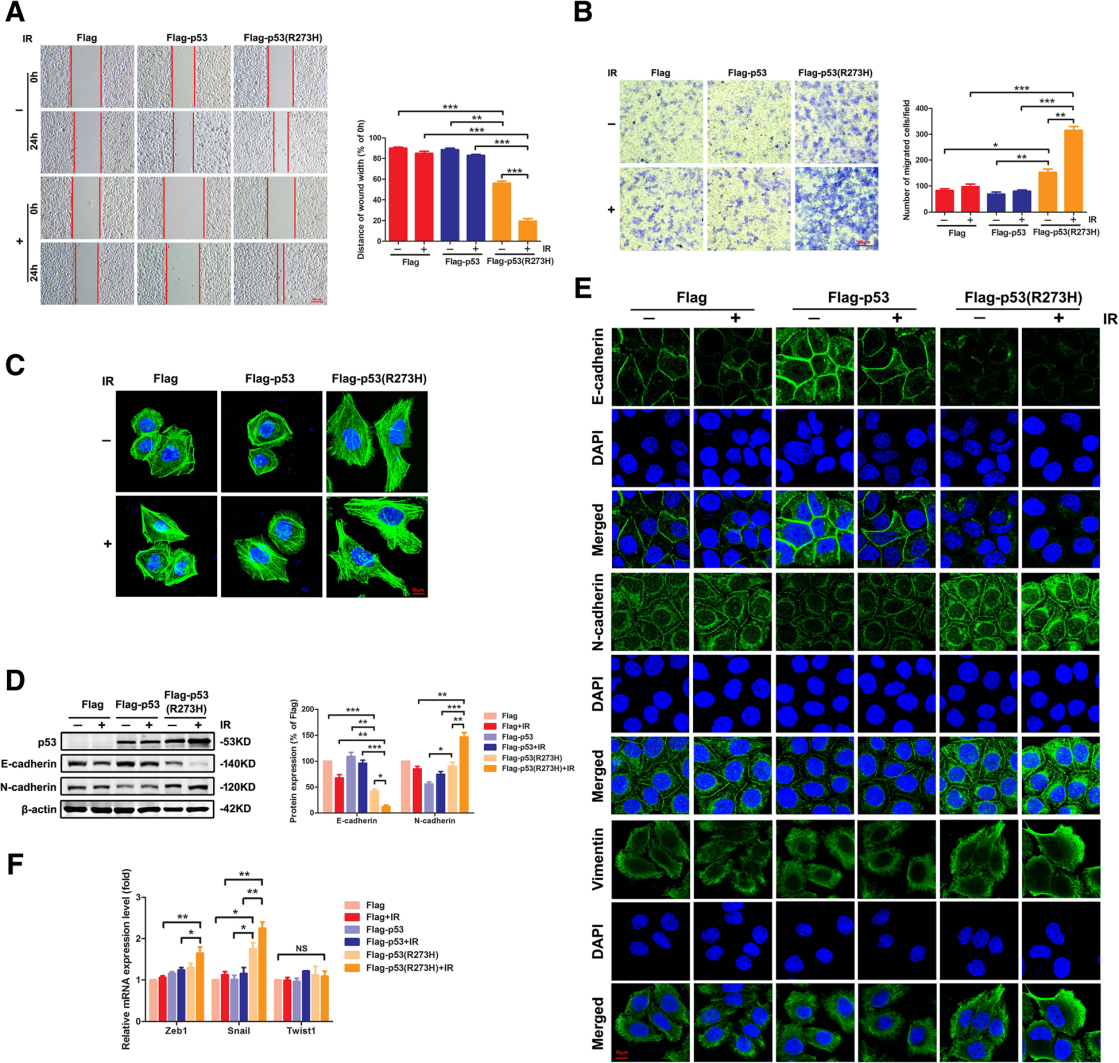
IR promoted cell motility and EMT in the presence of *mut-p53* in vitro. **a** A wound-healing assay was conducted to test the migration ability of cells. Wounds were set into a confluent cell layer of three *p53* status cell lines under IR or not. Photographs at time 0 and after 24 h were shown (left panel). Red bar, 100 μm. The distances of wound width were shown at the right panel. **b** The role of *p53* status in IR-induced invasion was demonstrated by transwell assay in H1299 cells (left panel). Red bar, 100 μm. The number of migrated cells were shown (right panel). **c** Cells were stained with phalloidin to examine F-actin stress fibers. Images were captured on a Zeiss fluorescence microscope. Red bar, 10 μm. **d** Effects of IR on E-cadherin and N-cadherin expressions in three *p53* status cell lines by western blotting analysis. **e** Immunofluorescence assay to determine the localization of EMT-associated proteins. Confocal microscopy images of E-cadherin, N-cadherin, Vimentin, and DAPI as “separate” or “merged” images. Red bar, 10 μm. **f** PCR assay analyzed the mRNA level of EMT-associated markers, Zeb1, Snail and twist1 in three *p53* status cell lines under IR or not. Results shown are the representative of three identical experiments. *P < 0.05, **P < 0.01, *** *P* < 0.001, Student’s t-test

According to the hot spots of *p53* gene mutation verified, vector, wildtype and the most hot mutant spot 273 of *p53* lentivirus were synthesized, and then transfected into the lung cancer H1299 cells (null of *p53*) to establish stable transfected cell clones respectively (p53 protein expression in these cells, Fig. 2d). The wound-healing assays were conducted on Flag cells, Flag-p53 cells and Flag-p53 (R273H) cells to detect the migration induced by IR (Fig. 2a). The results showed that without IR, the wound area of the Flag-p53 (R273H) cells healed obviously compared with the other two cells (*P* < 0.01). Notable, by irradiated 24 h, only the Flag-p53 (R273H) cells exhibited obviously healed capacity (*P* < 0.001). Following the above results, invasion of cells via transwell invasion experiment was observed. As shown, in comparison with Flag and Flag-p53 cells, the amount of Flag-p53 (R273H) cells transferring to the other surface of the membrane appeared more abundant. Moreover, this tendency was more significant under IR (*P* < 0.01, Fig. 2b). As acknowledged, the arrangement of the cytoskeleton was closely related to morphology and movement of cells [31, 32], FITC-Phalloidin staining was allowed to analyze the cytoskeleton of tumor cells. According to these results, a large number of microfilament cytoskeletons and pseudopodia appeared in the Flag-p53 (R273H) cells, especially under IR. In contrast, the Flag and Flag-p53 cells did not show any alteration of microfilament skeleton or evident presence of pseudopodia (Fig. 2**c**). It suggested that the invasion, migration, and motility of *mut-p53* cells could be improved especially under IR.

EMT process plays a vital role in tumor invasion and metastasis in various solid tumors, particularly, in NSCLC [33]. In order to study whether IR-induced tumor motility in presence of *mut-p53* was mediated by EMT, we evaluated the expression level of EMT markers of *mut-p53* cells after IR exposure by western blot. The results showed that E-cadherin level decreased significantly in Flag-p53 (R273H) cells (*P* < 0.05), while N-cadherin level increased (*P* < 0.01). Similarly, this tendency was more significant under IR. In Flag and Flag-p53 cells, there was no notable change of EMT marker proteins (Fig. 2d). Simultaneously, to further verify the data from western blot, we conducted immunofluorescence staining of cells in order to locate EMT marker and found the same results for E-cadherin and N-cadherin generated by western blotting assay. The EMT-associated proteins, vimentin, had also a clearly elevated expression in Flag-p53 (R273H) cells, and IR further promoted this expression (Fig. 2e). The transcriptional mechanisms for EMT have been well elucidated. The Snail transcription factor enhances expression of the EMT transcription factor zinc finger E-box binding homeobox 1 (Zeb1) and plays a role of master regulator of EMT [34]. PCR assays results showed the mRNA level of other EMT-associated markers, Snail and Zeb1 were both increased in Flag-p53 (R273H) cells, whereas Twist1 showed no change (Fig. 2f). Hence, IR promoted *mut-p53* GOF activity of tumor motility might be affected through the process of EMT.

### CTSL crucially mediated the IR-induced EMT in the presence of *mut-p53*

**Fig. 3:**
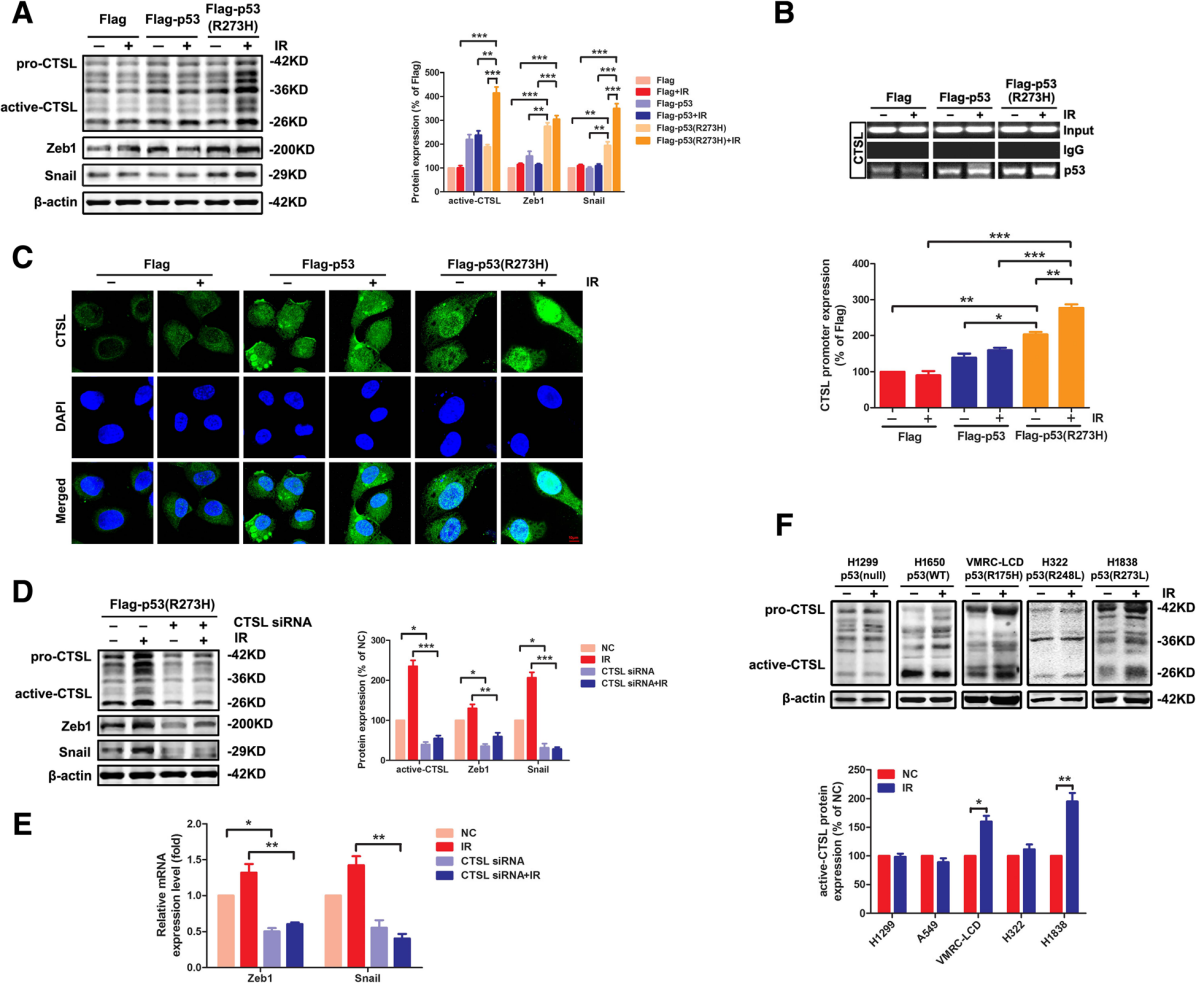
CTSL crucially mediated the IR-induced EMT in the presence of *mut-p53*. **a** The levels of CTSL and EMT-associated transcription factors were examined by western blotting in three *p53* status cell lines under IR or not (left panel). The corresponding quantities of protein expression were shown (right panel). **b** Cells were examined by ChIP assay with or without IR treatment. The recruitment of *p53* to the CTSL promoter was shown (up panel). The corresponding quantities of CTSL promoter expression were shown (down panel). **c** Expression and intracellular location of CTSL in three *p53* status cell lines under/ or not IR was determined by immunofluorescence assay. Red bar, 10 μm **d**) Effects of CTSL siRNA and IR on EMT-associated Zeb1 and Snail protein expressions in Flag-p53 (R273H) H1299 cells by western blotting analysis. **e**) PCR assay was used to analyze the mRNA level of Zeb1 and Snail in Flag-p53 (R273H) H1299 cells with or without IR. **f**) The expression of CTSL was analyzed by western blotting in various lung cancer cells with the relatively common mutation spot 175, 248 and 273 of *p53*. Data are shown as mean ± S.D., *n* = 3, *P < 0.05, **P < 0.01, *** *P* < 0.001, Student’s t-test

To further investigate the molecular mechanism of IR promoting the EMT in presence of *mut-p53*, Flag cells, Flag-p53 cells, and Flag-p53 (R273H) cells were analyzed by western blotting with or without IR treatment. The results showed that the expression of EMT-associated transcription factors Snail and Zeb1 was actually increased in Flag-p53 (R273H) cells compared to the other cell lines (*P* < 0.01), besides the expression of CTSL. Notable, the expression of CTSL was elevated in Flag-p53 cells and Flag-p53 (R273H) cells, which was in accordance with the conclusion that CTSL may be the target protein of p53 [35, 36]. Interestingly, the expression of CTSL was increased remarkably by IR only in Flag-p53 (R273H) cells (*P* < 0.001, Fig. 3a). The irradiation doses were investigated in Additional file 6: Figure S1, and the expression of CTSL peaked at 8 Gy, which was in keeping with our previous report [26]. ChIP assay was used to further demonstrate the interaction of *p53* and CTSL. The CTSL promoter activity of promoter region (− 192/17 bp, Additional file 7: Figure S2A) clearly indicated the presence of *p53* binding site within that region. The results showed that both *wt-p53* and *mut-p53* (R273H) could bind to the promoter of CTSL. The combining capacity of wild-type *p53* to the promoter of CTSL was similar with/without IR while for *mut-p53* (R273H) was significantly enhanced after IR (*P* < 0.01, Fig. 3b), in conformity with the western blotting results. This data strongly suggested that the *mut-p53* should act on a specific target that tremendously elevating the expression or function of CTSL under IR. To elucidate whether *p53* regulates the expression and function of CTSL, we next identified immunofluorescence staining of CTSL in these cells. The expressions of CTSL in both Flag-p53 cells and Flag-p53 (R273H) cells were increased compared with Flag cells. However, CTSL in Flag-p53 (R273H) cells could be transported from the cytoplasm into the nucleus after irradiation, but not in Flag-p53 group (Fig. 3c). Importantly, only CTSL located in the nucleus could play an important role in regulating cellular transcription factors and thus affecting the progression of cancers [37]. Further, the CTSL siRNA was used in Flag-p53 (R273H) cells, western blotting showed CTSL siRNA could effectively inhibit the expression of CTSL (*P* < 0.05), even under IR (*P* < 0.001). The protein and mRNA level of Snail and Zeb1 were both reduced by CTSL siRNA (Fig. 3d and e). In order to further verify the interaction of *p53* and CTSL, various lung cancer cell lines with different mutation spot 175, 248 and 273 of *p53* were treated with IR. As shown in western blotting, the expression of CTSL was increased remarkably by IR only in VMRC-LCD (p53-R175H) (*P* < 0.05) and H1838 (p53-R273L) (*P* < 0.01) cells (Fig. 3f). This result was confirmed again in H1299 cells transfected with the mutation spot 175, 248 and 273 of *p53* plasmid DNA, respectively (Additional file 8: Figure S3). In view of these findings, CTSL was the key target for the GOF of *mut-p53* by promoting the EMT especially underwent IR.

### The *mut-p53* regulates the transcription of CTSL depended on Egr-1 activation under IR

**Fig. 4:**
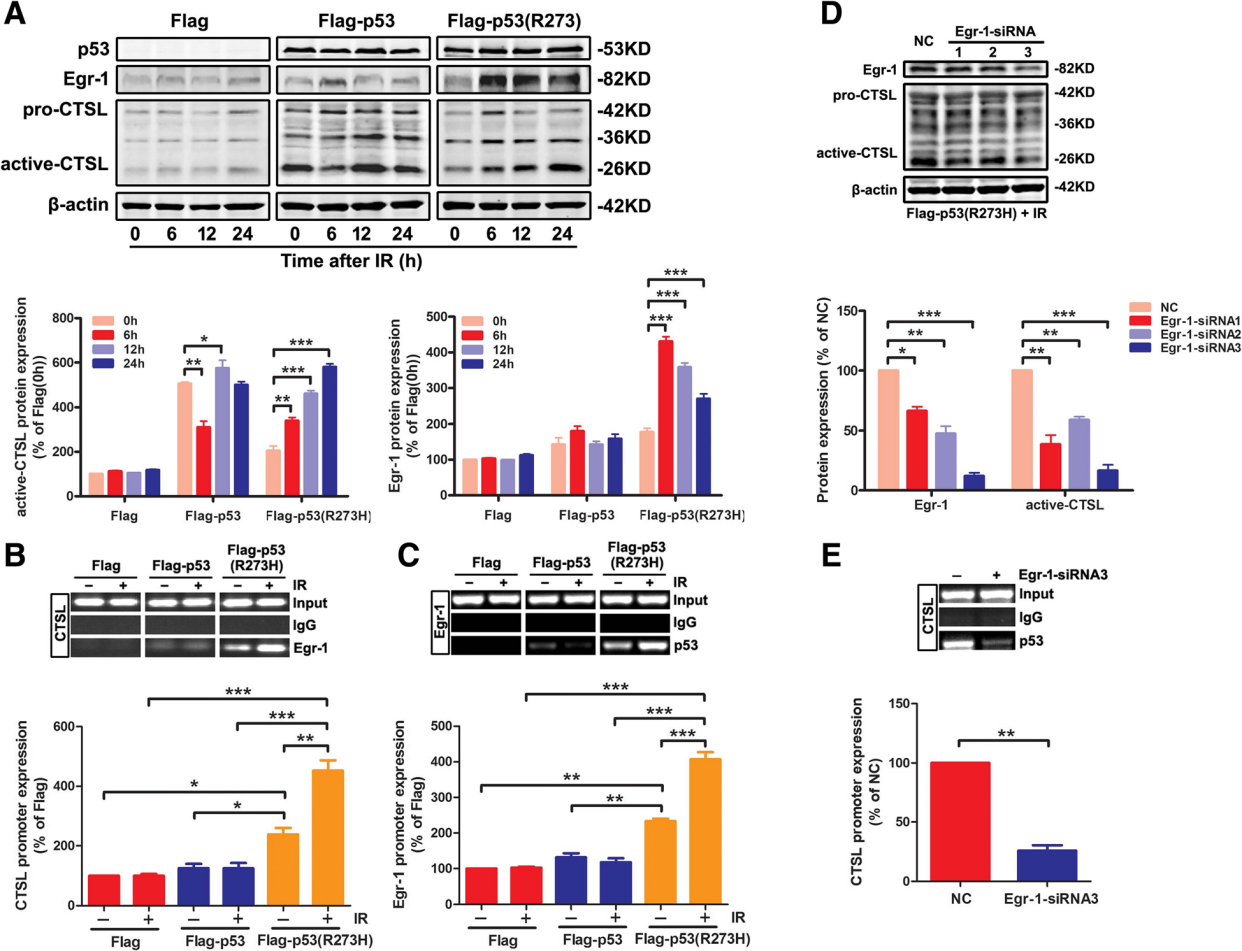
The *mut-p53* regulates the transcription of CTSL depended on Egr-1 activation under IR. **a** Cells were treated with/ without IR at various time points (0, 6, 12, 24 h). The levels of CTSL and Egr-1 were examined by western blotting (up panel). The corresponding quantities of protein expression were shown (down panel). **b** Cells were treated as mentioned above and harvested for ChIP assay to verify the interaction between Egr-1 and the promoter of CTSL under/ or not IR (up panel). The corresponding quantities of CTSL promoter expression were shown (down panel). **c** ChIP assay was analyzed to verify the interaction between *p53* and the promoter of Egr-1 in three *p53* status cell lines with or without IR treatment. The recruitment of *p53* to the Egr-1 promoter was shown (up panel). The corresponding quantities of Egr-1 promoter expression were shown (down panel). **d** Flag-p53 (R273H) cells were transfected with three Egr-1 si-RNA sequences under IR and total cell lysates were analyzed for Egr-1 and CTSL by western blotting using the indicated antibodies. **e** Flag-p53 (R273H) cells were transfected with Egr-1 si-RNA sequence under IR and harvested for ChIP assay to verify the interaction between *p53* and promoter of CTSL (up panel). The corresponding quantities of CTSL promoter expression were shown (down panel). Data are shown as mean ± S.D., *n* = 3, *P < 0.05, **P < 0.01, *** *P* < 0.001, Student’s t-test

As mentioned before, Egr-1, an immediate early gene and a zinc finger transcription factor closely related to the *p53*, was rapidly induced in response to IR [38]. Herein, we verified whether Egr-1 was the target that could promote *mut-p53* to bind to the promoter of CTSL under IR. The Flag cells, Flag-p53 cells and Flag-p53 (R273H) cells were treated with IR or not, at various time points (0, 6, 12, 24 h). Western blotting analysis showed that the expression of Egr-1 was higher in Flag-p53 (R273H) cells than in Flag or Flag-p53 cells. CTSL slightly decreased at 6 h after IR in Flag-p53 cells, but no change at 24 h compared to 0 h. Meanwhile, CTSL in Flag-p53 (R273H) cells was induced distinctly after IR (*P* < 0.001), and it was time-dependent peaking at 24 h. It was interesting that Egr-1 expression level peaked at 6 h in Flag-p53 (R273H) cells (*P* < 0.001), which was earlier than augment of CTSL expression in Flag-p53 group. Besides, there was no obvious alteration of Egr-1 expression appeared in the Flag or Flag-p53 cells. Thus, we speculated that *mut-p53* activated CTSL expression under IR and its activation was likely to be associated with the earlier-activated Egr-1 (Fig. 4a). ChIP assay was conducted to further investigate the relationship of Egr-1 with CTSL. Additional file 7: Figure S2A showed the Egr-1 binding site − 126/1 bp of CTSL promoter region. The PCR results showed that Egr-1 could bind to the promoter of CTSL upstream only in Flag-p53 (R273H) cells, and IR could enhance this interaction (Fig. 4b). We also detected an upstream region of the Egr-1 promoter and found binding sites (− 86/− 3 bp of Egr-1 promoter region, Additional file 7: Figure S2B) of *mut-p53*. Similarly, the *mut-p53* could bind to Egr-1 and IR also enhanced this interaction (Fig. 4c). This might be the root cause of *mut-p53* mediated CTSL activation. To strengthen that, VMRC-LCD (p53-R175H) and H1838 (p53-R273L) cells were treated with/ without IR and harvested for ChIP assay to verify the interaction between endogenous *mut-p53* and the promoter of CTSL. As show in Additional file 9: Figure S4A, both *mut-p53* (R175H) and *mut-p53* (R273L) could bind to the promoter of CTSL. Meanwhile the combining capacity of endogenous *mut-p53* to the promoter of CTSL was significantly enhanced after IR (*P* < 0.01). We further investigate the relationship of Egr-1 with CTSL promoter (Additional file 9: Figure S4B) and *mut-p53* with Egr-1 promoter (Additional file 9: Figure S4C) in these two cell lines. The results were in consistent with the *mut-p53* over-expressed cells. Accordingly, these results show that both endogenous *mut-p53* and over-expressed *mut-p53* are upstream regulators of CTSL.

In order to further determine whether Egr-1 mediated the transcriptional regulation of *mut-p53*/CTSL, three Egr-1 specific siRNA sequences were used to inhibit the expression of Egr-1 in Flag-p53 (R273H) cells with IR. Western blotting analysis showed that Egr-1-siRNA3 sequence could effectively inhibit the expression of Egr-1 (*P* < 0.001), and the CTSL expression was restrained obviously after IR (*P* < 0.01, Fig. 4d). Meanwhile, the *mut-p53* could not bind to the promoter of CTSL when Egr-1 gene has successfully interfered (*P* < 0.01, Fig. 4e). The results above showed that the *mut-p53* regulated the transcription of CTSL depended on Egr-1 activation under IR.

### CTSL was correlated with *p53* mutation and tumor malignancy in NSCLC patients

**Fig. 5:**
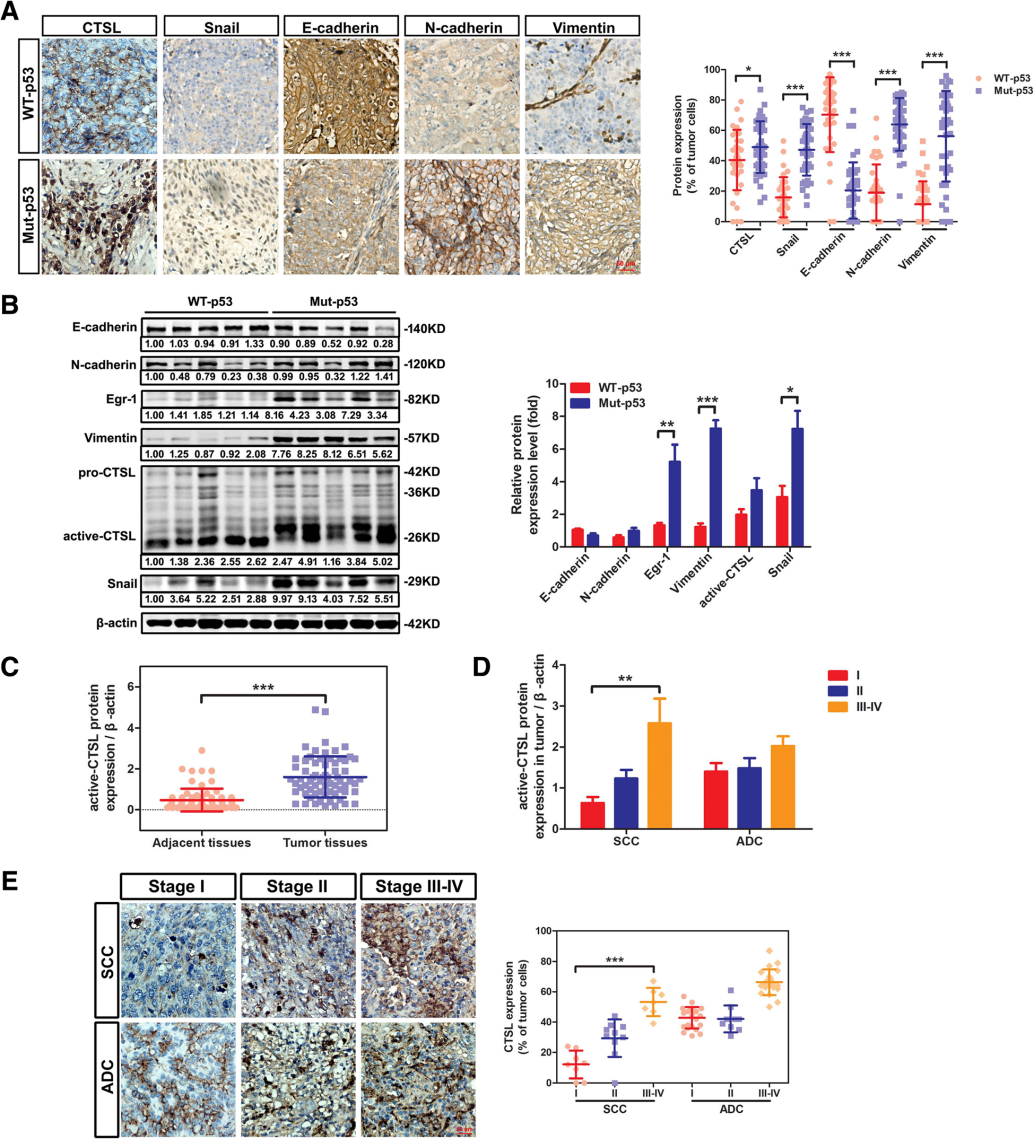
CTSL was correlated with *p53* mutation and tumor malignancy in NSCLC patients. **a** The expression of CTSL and EMT markers were examined in *mut-p53* NSCLC tissues (*n* = 35) and *wt-p53* NSCLC tissues (*n* = 43) using immunohistochemistry (representative images were shown in the left panel). The corresponding quantities of protein expression were shown (right panel). **b** Four tumor tissues were randomly selected respectively from the *wt-p53* group and *mut-p53* group for western blotting analysis of CTSL, Egr-1 and EMT markers (left panel). Band intensity of proteins was quantified and relative-fold change with *mut-p53* vs. *wt-p53* is presented (right panel). **c** Western blotting analysis of CTSL expression was different between tumor tissues and paired adjacent tissues. **d** The expression of CTSL progressively increased with increasing malignancy grade in squamous cell carcinoma but not evident in adenocarcinoma using western blotting analysis. **e** Immunohistochemical staining was conducted to examine the expression of CTSL in lung cancer tissues with I-IV malignant grades (representative images were shown in left panel). The correlation between CTSL and malignant grades was shown (right panel). Data are shown as mean ± S.D., n = 3, *P < 0.05, **P < 0.01, *** *P* < 0.001, Student’s t-test

IHC analyses were performed on 78 cases of paraffin-embedded lung cancer tissues. We found that CTSL and EMT markers (Snail, E-cadherin, N-cadherin, and Vimentin) were correlated with *p53* mutation in NSCLC tissues (*P* < 0.05). Interestingly, CTSL in *mut-p53* tissues was mainly located in the nucleus, while when in *wt-p53* tissues could be observed in the cytoplasm (Fig. 5a). It was indicated that *mut-p53* induced the EMT of NSCLC through promoting the transport of CTSL from the cytoplasm into the nucleus. Similarly, western blotting analysis demonstrated that N-cadherin, Vimentin (*P* < 0.001) and Snail (*P* < 0.05) were up-regulated in *mut-p53* group, and E-cadherin was down-regulated. Moreover, as with CTSL, Egr-1 (*P* < 0.01) was elevated in *mut-p53* group, indicating *mut-p53* promoted CTSL through Egr-1 (Fig. 5b). Therefore, both CTSL and Egr-1 play important roles in the *mut-p53* mediated EMT of NSCLC.

To better understanding the effect of CTSL on tumor progression, a western blotting analysis was conducted on NSCLC tissues (Additional file 10: Figure S5). As was shown, CTSL was high-expressed in most tumor tissues compared with adjacent tissues (*P* < 0.001, Fig. 5c). Additionally, the expression of CTSL progressively increased with increasing malignancy grade in squamous cell carcinoma (SCC) (*P* < 0.01), while not evident in adenocarcinoma (ADC) (Fig. 5d). Furthermore, immunohistochemistry analysis showed CTSL expression was higher in stage III and stage IV of NSCLC (Fig. 5e). Thus, high expression of CTSL was also associated with tumor progression.

### CTSL promoted IR-induced EMT in the presence of *mut-p53* in vivo

**Fig. 6:**
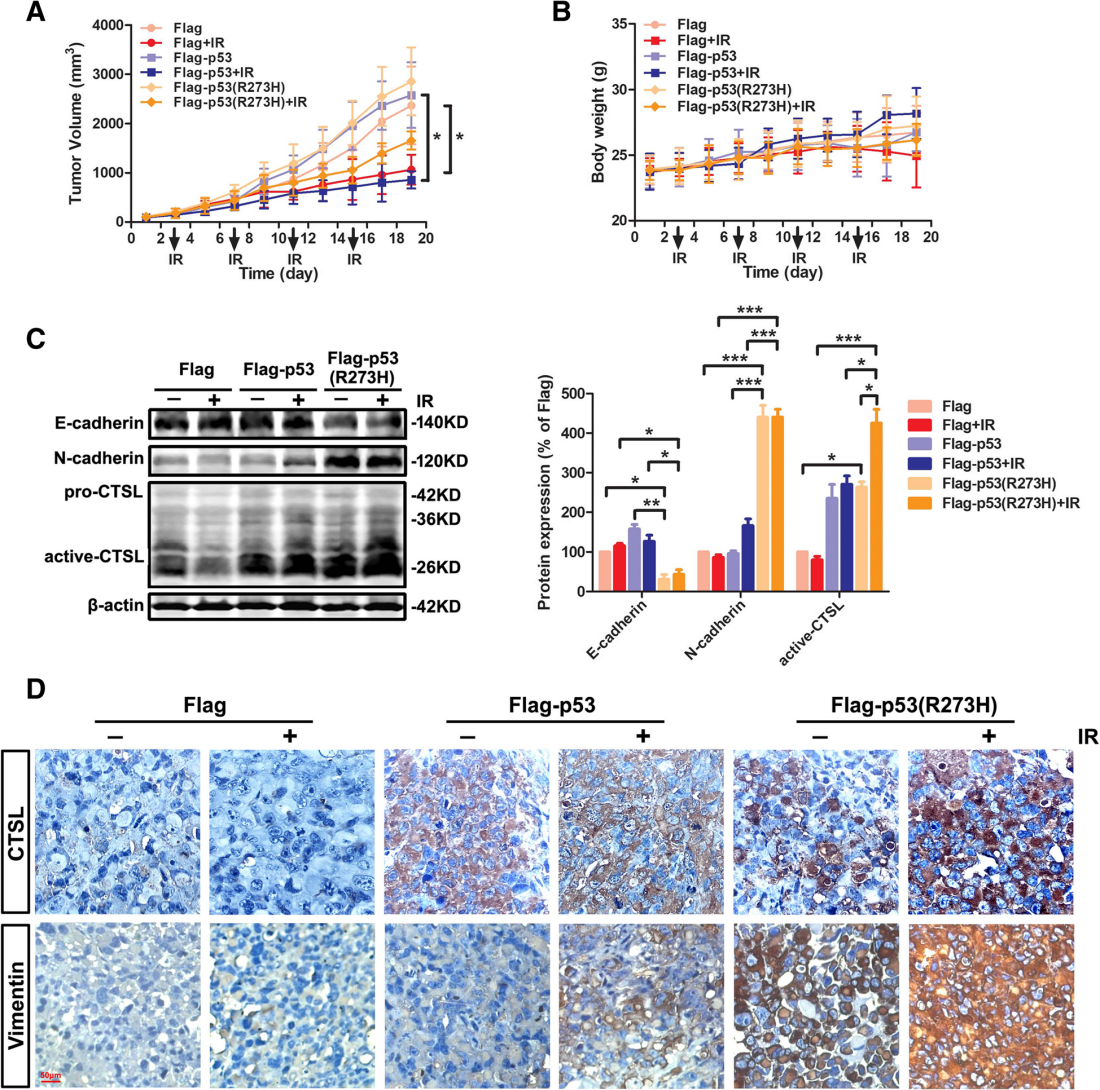
CTSL promoted IR-induced EMT in the presence of *mut-p53* in vivo. **a** and **b**) Subcutaneous tumor xenograft models established using Flag, Flag-p53, and Flag-p53 (R273H) cells when irradiated or not. The tumor volumes were measured and the nude mice were weighed every 2 days until the mice were killed. The curves were shown respectively. **c** The tumors were collected and processed for Western blot analysis of E-cadherin, N-cadherin, and CTSL (left panel). The corresponding quantities of protein expression were shown (right panel). **d** Immunohistochemical staining was conducted to verify the expression and cellular location of CTSL and Vimentin in tumor tissues. Data were shown as mean ± S.D., n = 3, *P < 0.05, **P < 0.01, *** *P* < 0.001, Student’s t-test

The encouraging results of previous studies indicate that CTSL regulated by *mut-p53* could dramatically promote EMT in *mut-p53* lung cancer cells after IR. We further investigated the effect of CTSL on EMT induced by IR in the presence of *mut-p53* in vivo. The subcutaneous tumor xenograft model was established using Flag cells, Flag-p53 cells, Flag-p53 (R273H) cells, and then treated or not with IR. As shown in Fig. 6a, tumor volume was 2369.66 ± 788.70 mm^3^ in the Flag group, 1066.16 ± 303.35 mm^3^ in Flag plus IR group, 2580.77 ± 664.94 mm^3^ in Flag-p53 group, 859.06 ± 176.12 mm^3^ in Flag-p53 plus IR group, 2857.55 ± 686.17 mm^3^ in Flag-p53 (R273H) group, and 1658.13 ± 183.92 mm^3^ in Flag-p53 (R273H) plus IR group. The average volumes of tumors were all inhibited by 20 Gy total dose of IR, and the radiotherapy showed effective therapeutic outcome both in Flag plus IR group (*P* < 0.05) and Flag-p53 plus IR group (*P* < 0.05). The weights of mice were also recorded (Fig. 6b). As was shown, there was no significant difference among those groups in tumor weight. Western blotting analysis of subcutaneous tumors showed that E-cadherin was significantly inhibited in Flag-p53 (R273H) group (*P* < 0.05) or Flag-p53 (R273H) plus IR group (*P* < 0.05), while the N-cadherin was activated (*P* < 0.001) (Fig. 6c). Meanwhile, the locations of CTSL and Vimentin in tumor cells were investigated by immunohistochemical staining. Nuclear CTSL and Vimentin were remarkably increased in Flag-p53 (R273H) plus IR group compared to Flag-p53 plus IR group, which was consistent with the results of experiments in vitro (Fig. 6d). The results of tumor xenograft experiment confirmed that although IR could reduce the tumor growth, it also promotes the EMT of the cancer cells in the presence of *mut-p53*, owing to increase the expression of its downstream protein CTSL.

## Discussion

**Fig. 7:**
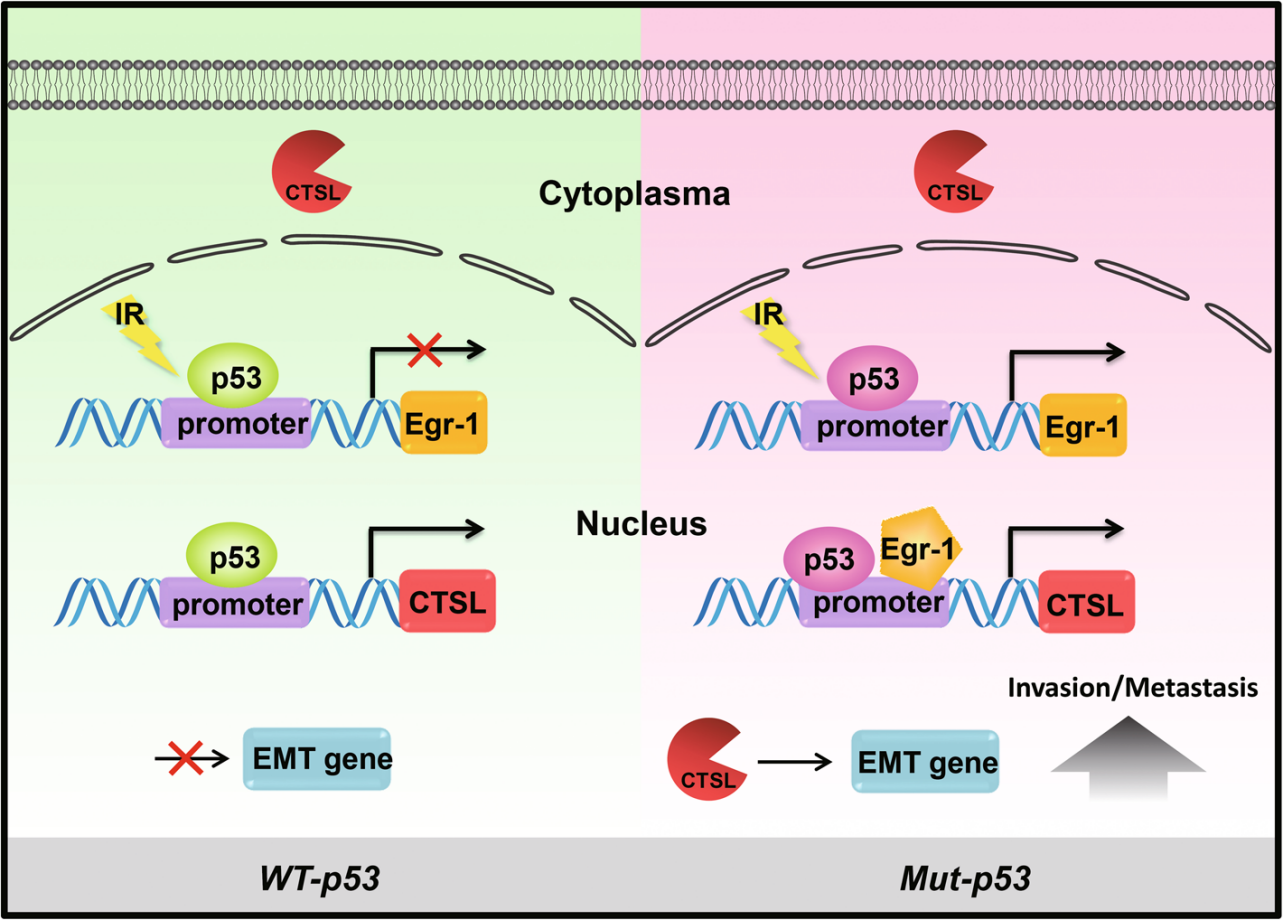
Schematic diagram of the mechanism by which CTSL promotes IR-induced EMT of lung cancer via the *mut-p53*/Egr-1 signaling pathway. Only mutated *p53* stimulates Egr-1 expression under IR. Then, mutated *p53* and Egr-1 synergistically stimulate CTSL transcription. Finally, nuclear CTSL directly upregulated EMT genes to enhance invasion and metastasis in *mut-p53* lung cancer cells

It is well known that radiotherapy is an effective treatment of cancer, but also has different therapeutic effects on different cancers [39]. Emerging evidence demonstrates that IR can promote EMT process of cancer, mainly resulting in tumor metastasis [40]. Moreover, *p53* mutation, occurring in most cancer patients, may play a key role in the EMT process of tumor cells [8]. However, unfortunately, there is no evidence that addresses the relationship between *p53* gene status and IR-induced EMT, nor the mechanism. In this study, we demonstrated, for the first time, that CTSL regulated by *mut-p53* was a key player in the IR-induced EMT of NSCLC and provide direct molecular mechanistic insights into precisely how this gene regulates IR-induced EMT (Fig. 7).

An important finding of this study was that the patients with the *p53* mutation were more likely to have tumor metastasis, and the survival rate of IR-treated patients with *mut-p53* was significantly decreased. The hot mutant spots of *p53* mainly existed on the exon 5–8 and almost were point mutations. Among them, *p53* mutated at codon 273 played a vital role in metastasis, with a high metastasis rate of 40%. It was indicated that *p53* mutation (particularly in codon 273) could be regarded as a key indicator of tumor metastasis in NSCLC patients.

Tumor metastasis is a complex pathological process that requires the invasion and migration of tumor cells, which are closely related to the EMT-induced movement of the tumor [41–43]. It was demonstrated here that both, the migration ability and cell motility of *mut-p53* cells (codon 273), could be improved by IR. Furthermore, the EMT-associated proteins were also clearly elevated in *mut-p53* cells (codon 273), and IR further promotes these expressions. It was suggested that the metastasis of *p53*-mutated cells increases after IR, through the process of EMT, probably.

CTSL, the main member of the Cathepsins family, not only takes part in regulating invasion and metastasis of tumor [44, 45], but also modulates transcription of EMT related genes [12]. It was controversial that whether CTSL could be regulated by *mut-p53* in human glioblastoma cells [11, 18]. In this study, the expressions of CTSL were increased by *wt-p53* and remarkably upregulated by IR in *mut-p53* lung cancer cells. The up-regulated CTSL in *wt-p53* lung cancer cells was mainly mostly distributed in the cytoplasm, while CTSL could be transported from the cytoplasm into nucleus after treated with IR only in the *mut-p53* group, playing an important role in affecting the EMT of lung cancer cells. It is well explained that although the elevated expression of CTSL occurred in the *wt-p53* tumor, it had a weak influence on *p53* GOF activity of tumor motility. In addition, why the CTSL transcribed by *mut-p53* could be located in the nucleus, needs further investigation. We suspect that the mechanism may be closely related to the initiation site of *p53*-regulated CTSL translation [46]. Nevertheless, our previous hypothesis was confirmed, so that IR-activated transcription of CTSL was *mut-p53* dependent and affected the EMT of cancer cells.

Most importantly, our data discovered a novel mechanism of the CTSL transcription mediated by the *mut-p53* after IR. Firstly, the peaked expression level of Egr-1 in the *mut-p53* group was earlier than augment of CTSL expression. On the other hand, Egr-1 could bind to the promoter of CTSL upstream in the *mut-p53* group, but not in the *wt-p53* group. Similarly, the *p53* could bind to Egr-1 only in the *mut-p53* group with IR enhancing this interaction. Furthermore, CTSL expression in *p53*-mutated lung cancer cells was restrained obviously after IR by Egr-1 inhibition. Meanwhile, the *mut-p53* could not bind to the promoter of CTSL when Egr-1 gene has successfully interfered. The results showed that *mut-p53* regulates the transcription of CTSL depending on Egr-1 activation under IR, but not the *wt-p53*.

Moreover, our study further verified that CTSL expression level was significantly higher in tumor tissues than in adjacent tissues, and positively related to the tumor grade. Additionally, the expression of nuclear CTSL was dramatically elevated in *mut-p53* tissues, as well as EMT-associated proteins, which was in accordance with the result in lung cancer cells. Thus, CTSL can be regarded as a potential target in NSCLC patients.

In addition, the tumor formation of IR-treated groups was significantly reduced in the Flag and Flag-p53 nude mice, whereas the Flag-p53 (R273H) nude mice could not be evidently decreased. Importantly, after exposure to IR, CTSL nuclear location and EMT level of Flag-p53 (R273H) group were increased, consistent with the in vitro studies. It is worth noting that targeting CTSL might be very significant for conquering radiotherapy-induced EMT of lung cancer patients whose *p53* gene is mutant.

## Conclusions

Our studies indicated that *mut-p53* cells become invasive after IR, by a combination of Egr-1-dependent effects, including high levels of CTSL, Snail and Zeb-1 expression that leads to EMT behavior. Thus, CTSL acts not only as an additional diagnostic marker but also as a novel therapeutic target for NCSLC patients with *mut-p53*.

## Additional files

Additional file 1: **Table S1.** Clinicopathologic characteristics of NSCLC patients (DOCX 14 kb)

Additional file 2: **Table S2.** Primers for *p53 (DOCX 13 kb)*

Additional file 3: **Table S3.** Primers for Mutation (DOCX 13 kb)

Additional file 4: **Table S4.** Primers for PCR in ChIP assay (DOCX 13 kb)

Additional file 5: **Table S5.** Sequence for Egr-1 siRNA knockdown (DOCX 13 kb)

Additional file 6: **Figure S1.** The expression of CTSL peaked at 8 Gy in Flag-p53 (R273H) H1299 cells. Western blotting analysis of CTSL in Flag-p53 (R273H) H1299 cells under different irradiation dosages (up panel). Band intensity of active-CTSL was quantified and relative-fold change under IR vs. without IR is presented (down panel). Data were shown as mean ± S.D., *n* = 3, *, *P* < 0.05, **, *P* < 0.01, Student’s t-test. (JPG 154 kb)

Additional file 7: **Figure S2.** The sequence and position of the binding sites for the ChIP assay. A) Nucleotide sequences of the *p53* binding site (− 192/17 bp of CTSL promoter region) and Egr-1 binding site (− 126/1 bp of CTSL promoter region) aligned to homologous regions of the human CTSL genes. B) Schematic showed the Egr-1 promoter and the location of *p53* binding site (− 86/− 3 bp of Egr-1 promoter region). (JPG 1673 kb)

Additional file 8: **Figure S3.** The expression of CTSL significantly increased under IR in H1299 cells with the R175H and R273H mutation. Western blotting analysis of *p53* and CTSL in H1299 cells transfected with Flag, Flag-p53, Flag-p53 (R175H), Flag-p53 (R248Q) and Flag-p53 (R273H) plasmid respectively (up panel). Band intensity of active-CTSL was quantified and relative-fold change under IR vs. without IR is presented (down panel). Data were shown as mean ± S.D., n = 3, *, P < 0.05, ***, *P* < 0.001, Student’s t-test. (JPG 158 kb)

Additional file 9: **Figure S4.** The endogenous *mut-p53* regulates the transcription of CTSL under IR in two lung cancer cell lines. A) VMRC-LCD (p53-R175H) and H1838 (p53-R273L) cells were treated with/ without IR and harvested for ChIP assay to verify the interaction between endogenous *mut-p53* and the promoter of CTSL (up panel). The corresponding quantities of CTSL promoter expression were shown (down panel). B) Cells were treated as mentioned above and harvested for ChIP assay to verify the interaction between Egr-1 and the promoter of CTSL under/ or not IR (up panel). The corresponding quantities of CTSL promoter expression were shown (down panel). C) ChIP assay was analyzed to verify the interaction between *mut-p53* and the promoter of Egr-1 in two endogenous *mut-p53* cell lines with or without IR treatment. The recruitment of endogenous *mut-p53* to the Egr-1 promoter was shown (up panel). The corresponding quantities of Egr-1 promoter expression were shown (down panel). Data are shown as mean ± S.D., n = 3, *P < 0.05, **P < 0.01, *** *P* < 0.001, Student’s t-test. (JPG 632 kb)

Additional file 10: **Figure S5.** The levels of the CTSL protein were examined in NSCLC tissues using western blotting. Band intensity of active-CTSL was quantified and relative-fold change was presented. (JPG 985 kb)

## Abbreviations

ADC: adenocarcinoma
BSA: bovine serum albumin
ChIP: Chromatin immunoprecipitation
CTSL: Cathepsin L
Egr-1: Early growth response protein-1
EMT: epithelial-mesenchymal transition
FBS: fetal bovine serum
GOF: gain-of-function
IR: Ionizing radiation
*mut-p53*: mutant *p53*
NSCLC: non-small cell lung cancer
PBS: phosphate buffer saline
SCC: squamous cell carcinoma
*wt-p53*: wild-type *p53*
Zeb1: zinc finger E-box binding homeobox 1
