# Supplementary material for: Cathepsin L activated by mutant p53 and Egr-1 promotes ionizing radiation-induced EMT in human NSCLC
Source: J Exp Clin Cancer Res. 2019 Feb 7;38:61. doi: 10.1186/s13046-019-1054-x (PMC6367810; doi:10.1186/s13046-019-1054-x)
Supplement: Supplementary file 1 — Table S1. Clinicopathologic characteristics of NSCLC patients (DOCX 14 kb) [file 13046_2019_1054_MOESM1_ESM.docx]

**Table S1: Clinicopathologic characteristics of NSCLC patients**

| Clinicopathological feature | n |
| --- | --- |
|  |  |
| Gender |  |
| Male | 48 |
| Female | 30 |
| Age (years) |  |
| <60 | 29 |
| ≥60 | 49 |
| Histological type |  |
| SCC | 25 |
| ADC | 53 |
| Differentiation |  |
| well | 9 |
| Moderate | 47 |
| poor | 22 |
| T stage |  |
| T1a-b | 19 |
| T2a-b | 43 |
| T3-4 | 16 |
| N stage |  |
| N0 | 45 |
| N1-3 | 33 |
| M stage |  |
| M0 | 74 |
| M1 | 4 |
| TNM stage |  |
| I | 31 |
| II | 19 |
| III-IV | 28 |
