# Supplementary material for: Cathepsin L activated by mutant p53 and Egr-1 promotes ionizing radiation-induced EMT in human NSCLC
Source: J Exp Clin Cancer Res. 2019 Feb 7;38:61. doi: 10.1186/s13046-019-1054-x (PMC6367810; doi:10.1186/s13046-019-1054-x)
Supplement: Supplementary file 2 — Table S2. Primers for p53 (DOCX 13 kb) [file 13046_2019_1054_MOESM2_ESM.docx]

**Table S2: Primers for *p53***

| Region | Primer | Primer sequence |
| --- | --- | --- |
| *p53* (exons 5 to 8) | forward | 5′- tacggtttccgtctgggcttct-3′ |
|  | reverse | 5′- tgttgttgggcagtgctcgcttag -3′ |
