# Supplementary material for: Cathepsin L activated by mutant p53 and Egr-1 promotes ionizing radiation-induced EMT in human NSCLC
Source: J Exp Clin Cancer Res. 2019 Feb 7;38:61. doi: 10.1186/s13046-019-1054-x (PMC6367810; doi:10.1186/s13046-019-1054-x)
Supplement: Supplementary file 3 — Table S3. Primers for Mutation (DOCX 13 kb) [file 13046_2019_1054_MOESM3_ESM.docx]

**Table S3: Primers for Mutation**

| Mutant region | Primer | Primer sequence |
| --- | --- | --- |
| Flag-p53(R175H) | forward | 5′- cactgcccccaccatgagcgc -3′ |
|  | reverse | 5′- gtgtactgcctccaacactcc -3′ |
| Flag-p53(R248Q) | forward | 5′- cagaggcccatcctcaccatc -3′ |
|  | reverse | 5′- aggacgtacccgccgtacttg -3′ |
| Flag-p53(R273H) | forward | 5′- catgtttgtgcctgtcctggg-3′ |
|  | reverse | 5′- cctgccttgtcgaaactccac -3′ |
