# Supplementary material for: Cathepsin L activated by mutant p53 and Egr-1 promotes ionizing radiation-induced EMT in human NSCLC
Source: J Exp Clin Cancer Res. 2019 Feb 7;38:61. doi: 10.1186/s13046-019-1054-x (PMC6367810; doi:10.1186/s13046-019-1054-x)
Supplement: Supplementary file 4 — Table S4. Primers for PCR in ChIP assay (DOCX 13 kb) [file 13046_2019_1054_MOESM4_ESM.docx]

**Table S4: Primers for PCR in ChIP assay**

| Region | Primer | Primer sequence |
| --- | --- | --- |
| Cathepsin L（p53） | forward | 5′- catgcccggggcaccagctctg -3′ |
|  | reverse | 5′- ttcgcctgactctgcttcta -3′ |
| Cathepsin L（Egr-1） | forward | 5′- gtaaacaagccacgaaccgc -3′ |
|  | reverse | 5′- tctaaaacctcgggtctggg -3′ |
| Egr-1（p53） | forward | 5′- catgtacgtcacgacggagg-3′ |
|  | reverse | 5′- ctgggatctctcgcgactc -3′ |
