# Supplementary material for: Cathepsin L activated by mutant p53 and Egr-1 promotes ionizing radiation-induced EMT in human NSCLC
Source: J Exp Clin Cancer Res. 2019 Feb 7;38:61. doi: 10.1186/s13046-019-1054-x (PMC6367810; doi:10.1186/s13046-019-1054-x)
Supplement: Supplementary file 5 — Table S5. Sequence for Egr-1 siRNA knockdown (DOCX 13 kb) [file 13046_2019_1054_MOESM5_ESM.docx]

**Table S5: Sequence for Egr-1 siRNA knockdown**

| siRNA |  | Base sequence |
| --- | --- | --- |
| Si-Egr-1^698^ | sense | 5′- CCAACAGUGGCAACACCUUTT -3′ |
|  | anti-sense | 5′- AAGGUGUUGCCACUGUUGGTT -3′ |
| Si-Egr-1^1508^ | sense | 5′- GGCAUACCAAGAUCCACUUTT -3′ |
|  | anti-sense | 5′- AAGUGGAUCUUGGUAUGCCTT -3′ |
| Si-Egr-1^1819^ | sense | 5′- GCUGUCACCAACUCCUUCATT -3′ |
|  | anti-sense | 5′- UGAAGGAGUUGGUGACAGCTT -3′ |
| Negative control | sense | 5′- UUCUCCGAACGUGUCACGUTT -3′ |
|  | anti-sense | 5′- ACGUGACACGUUCGGAGAATT -3′ |
