# Supplementary figures and images for: Cathepsin L activated by mutant p53 and Egr-1 promotes ionizing radiation-induced EMT in human NSCLC
Source: J Exp Clin Cancer Res. 2019 Feb 7;38:61. doi: 10.1186/s13046-019-1054-x (PMC6367810; doi:10.1186/s13046-019-1054-x)

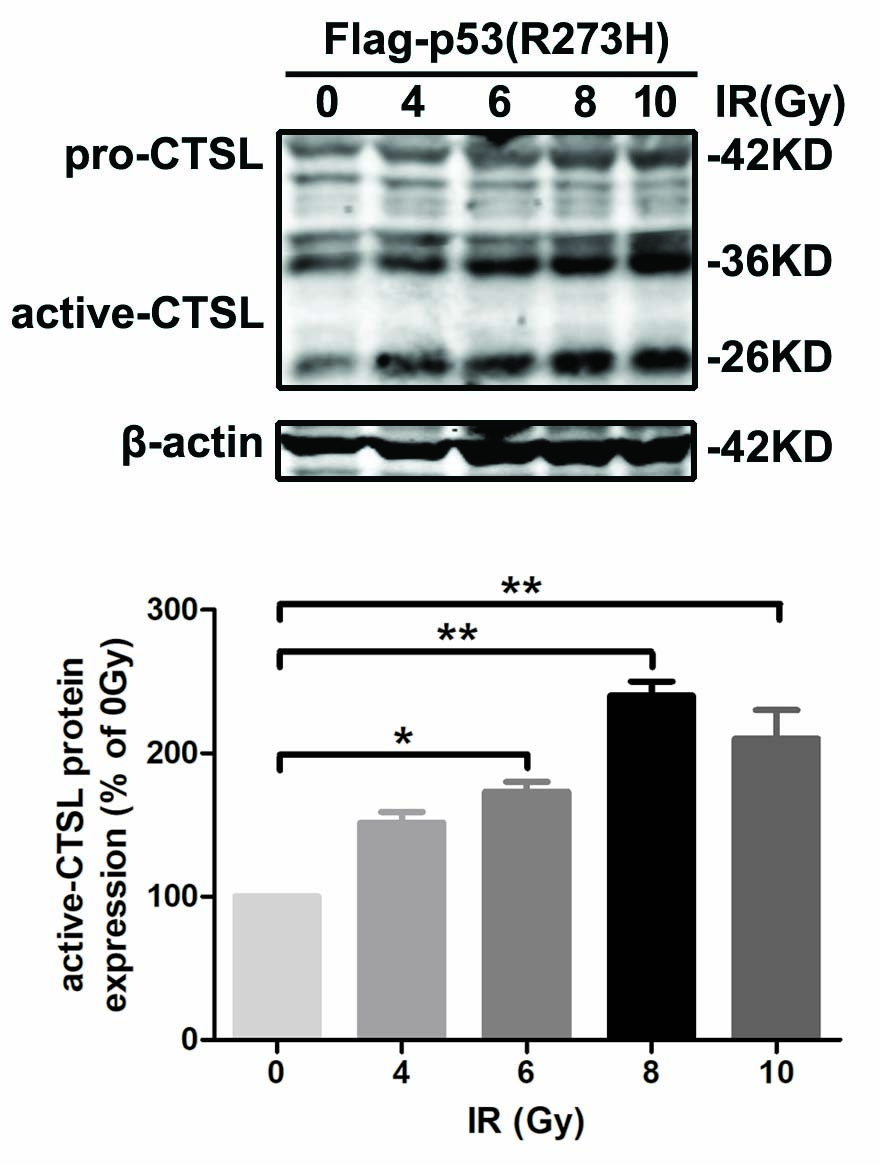

Supplement: Supplementary file 6 — Figure S1. The expression of CTSL peaked at 8 Gy in Flag-p53 (R273H) H1299 cells. Western blotting analysis of CTSL in Flag-p53 (R273H) H1299 cells under different irradiation dosages (up panel). Band intensity of active-CTSL was quantified and relative-fold change under IR vs. without IR is presented (down panel). Data were shown as mean ± S.D., n = 3, *, P < 0.05, **, P < 0.01, Student’s t-test. (JPG 154 kb) [file 13046_2019_1054_MOESM6_ESM.jpg]

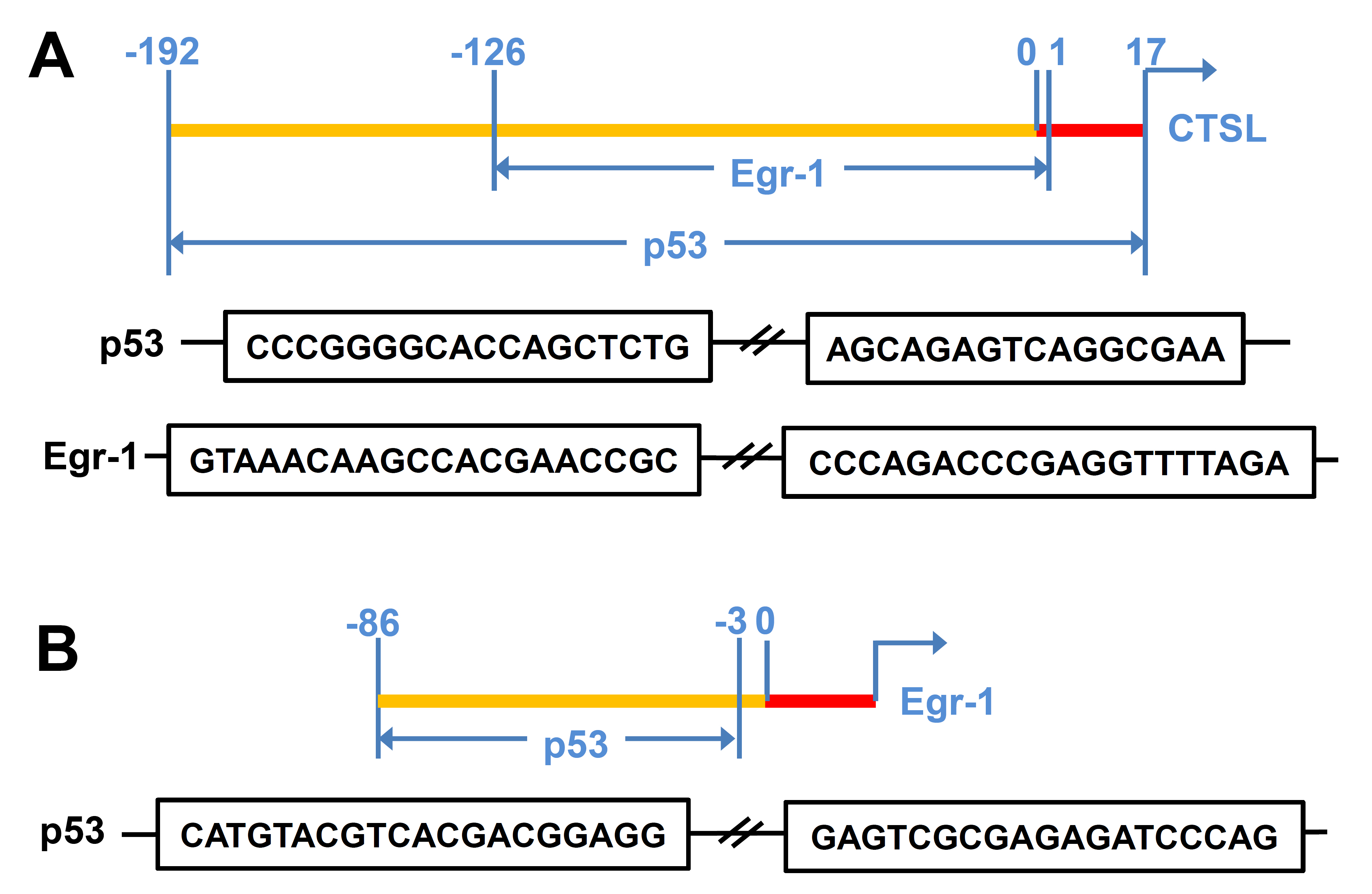

Supplement: Supplementary file 7 — Figure S2. The sequence and position of the binding sites for the ChIP assay. A) Nucleotide sequences of the p53 binding site (− 192/17 bp of CTSL promoter region) and Egr-1 binding site (− 126/1 bp of CTSL promoter region) aligned to homologous regions of the human CTSL genes. B) Schematic showed the Egr-1 promoter and the location of p53 binding site (− 86/− 3 bp of Egr-1 promoter region). (JPG 1673 kb) [file 13046_2019_1054_MOESM7_ESM.jpg]

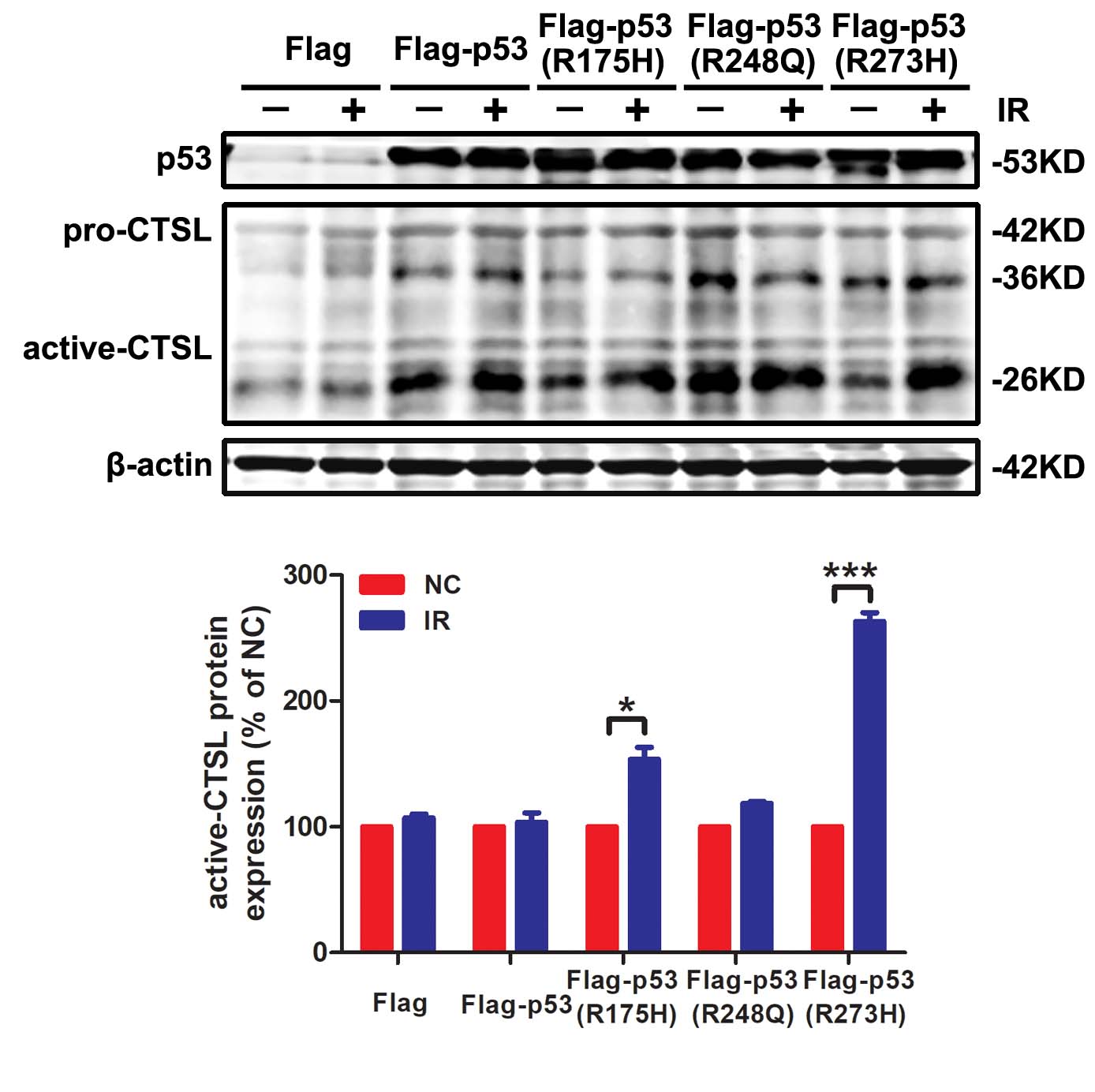

Supplement: Supplementary file 8 — Figure S3. The expression of CTSL significantly increased under IR in H1299 cells with the R175H and R273H mutation. Western blotting analysis of p53 and CTSL in H1299 cells transfected with Flag, Flag-p53, Flag-p53 (R175H), Flag-p53 (R248Q) and Flag-p53 (R273H) plasmid respectively (up panel). Band intensity of active-CTSL was quantified and relative-fold change under IR vs. without IR is presented (down panel). Data were shown as mean ± S.D., n = 3, *, P < 0.05, ***, P < 0.001, Student’s t-test. (JPG 158 kb) [file 13046_2019_1054_MOESM8_ESM.jpg]

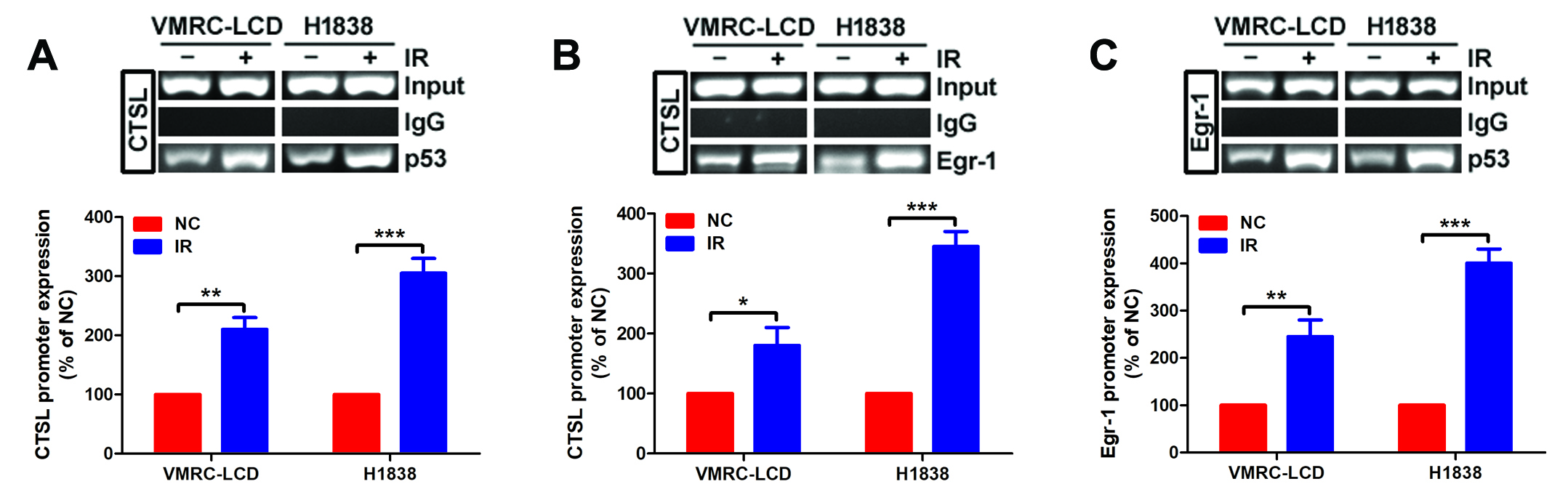

Supplement: Supplementary file 9 — Figure S4. The endogenous mut-p53 regulates the transcription of CTSL under IR in two lung cancer cell lines. A) VMRC-LCD (p53-R175H) and H1838 (p53-R273L) cells were treated with/ without IR and harvested for ChIP assay to verify the interaction between endogenous mut-p53 and the promoter of CTSL (up panel). The corresponding quantities of CTSL promoter expression were shown (down panel). B) Cells were treated as mentioned above and harvested for ChIP assay to verify the interaction between Egr-1 and the promoter of CTSL under/ or not IR (up panel). The corresponding quantities of CTSL promoter expression were shown (down panel). C) ChIP assay was analyzed to verify the interaction between mut-p53 and the promoter of Egr-1 in two endogenous mut-p53 cell lines with or without IR treatment. The recruitment of endogenous mut-p53 to the Egr-1 promoter was shown (up panel). The corresponding quantities of Egr-1 promoter expression were shown (down panel). Data are shown as mean ± S.D., n = 3, *P < 0.05, **P < 0.01, *** P < 0.001, Student’s t-test. (JPG 632 kb) [file 13046_2019_1054_MOESM9_ESM.jpg]

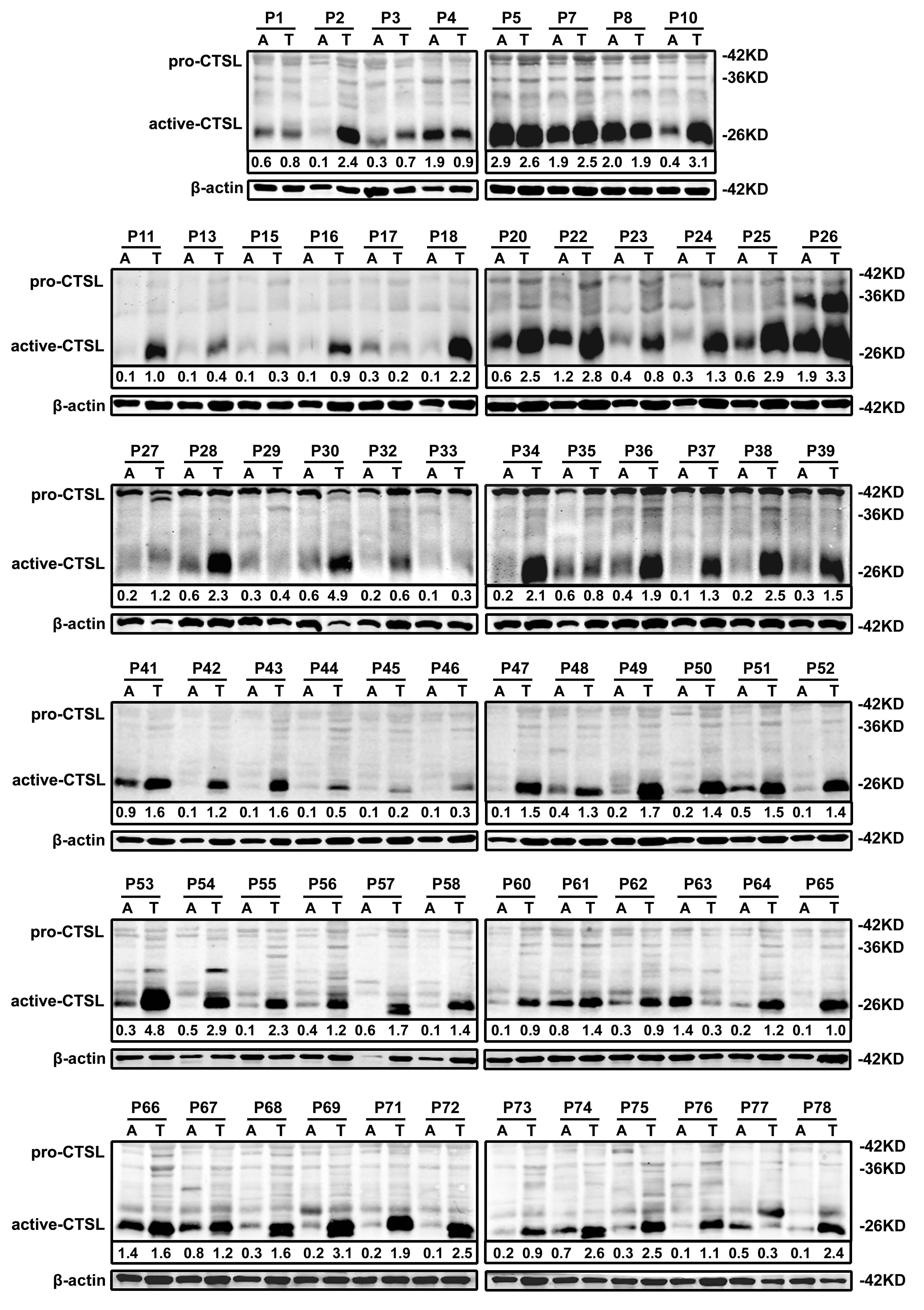

Supplement: Supplementary file 10 — Figure S5. The levels of the CTSL protein were examined in NSCLC tissues using western blotting. Band intensity of active-CTSL was quantified and relative-fold change was presented. (JPG 985 kb) [file 13046_2019_1054_MOESM10_ESM.jpg]
